# A Routing Protocol for Multisink Wireless Sensor Networks in Underground Coalmine Tunnels

**DOI:** 10.3390/s16122032

**Published:** 2016-11-30

**Authors:** Xu Xia, Zhigang Chen, Hui Liu, Huihui Wang, Feng Zeng

**Affiliations:** 1 School of Software, Central South University, Changsha 410075, China; wuwuxuxu@163.com (X.X.); HuiLiu@MissouriState.edu (H.L.); fengzeng@csu.edu.cn (F.Z.); 2 Hunan Vocational College of Security Technology, Changsha 410151, China; 3 Computer Science Department, Missouri State University, Springfield, MO 65897, USA; 4 Department of Engineering, Jacksonville University, 2800 University Blvd N, Jacksonville, FL 32211, USA; hwang1@ju.edu

**Keywords:** multisink, power control, coalmine tunnels, routing protocol

## Abstract

Traditional underground coalmine monitoring systems are mainly based on the use of wired transmission. However, when cables are damaged during an accident, it is difficult to obtain relevant data on environmental parameters and the emergency situation underground. To address this problem, the use of wireless sensor networks (WSNs) has been proposed. However, the shape of coalmine tunnels is not conducive to the deployment of WSNs as they are long and narrow. Therefore, issues with the network arise, such as extremely large energy consumption, very weak connectivity, long time delays, and a short lifetime. To solve these problems, in this study, a new routing protocol algorithm for multisink WSNs based on transmission power control is proposed. First, a transmission power control algorithm is used to negotiate the optimal communication radius and transmission power of each sink. Second, the non-uniform clustering idea is adopted to optimize the cluster head selection. Simulation results are subsequently compared to the Centroid of the Nodes in a Partition (CNP) strategy and show that the new algorithm delivers a good performance: power efficiency is increased by approximately 70%, connectivity is increased by approximately 15%, the cluster interference is diminished by approximately 50%, the network lifetime is increased by approximately 6%, and the delay is reduced with an increase in the number of sinks.

## 1. Introduction

Research on wireless communication technology in mine tunnels is very important because it is related to safety. To avoid monitoring system interruption caused by the breakage of communication cables as a result of accidents and to fit the cables for mobile work, the cable network should be replaced by the wireless network [1].

The main characteristics of a wireless sensor network (WSN) include flexible placement, simple extension, strong mobility, and self-organization. This makes the WSN extremely suitable for deployment in coalmine tunnels; thus, their deployment is of considerable current interest in the field of coalmine safety production [2].

**Figure 1:**
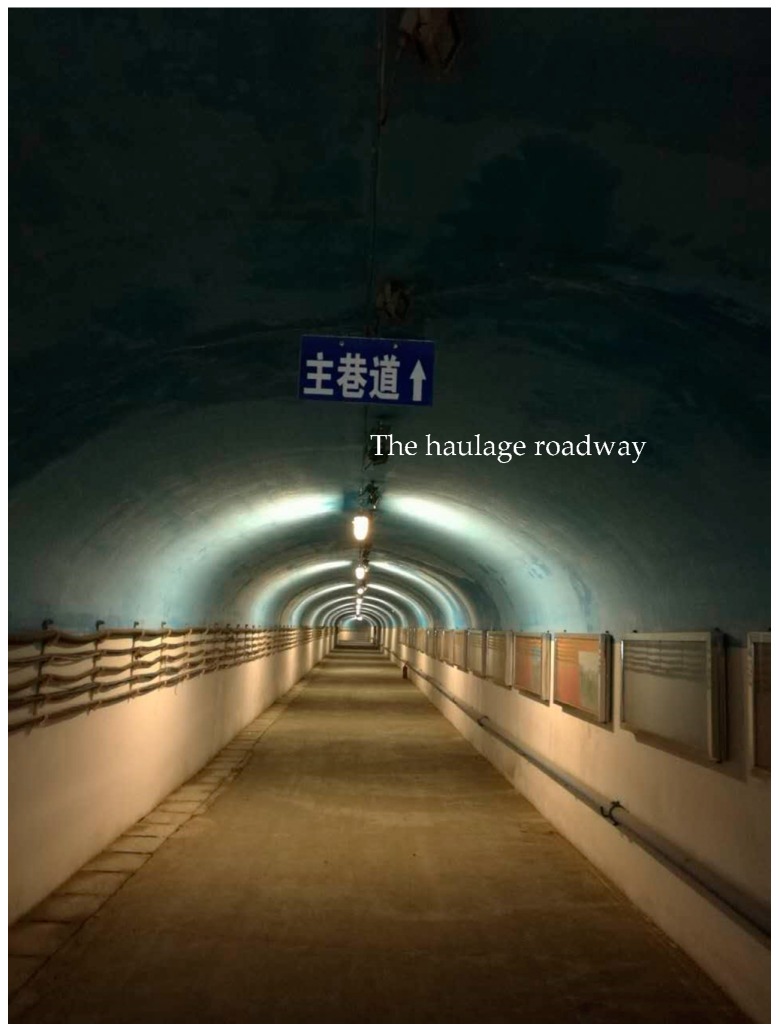
The haulage roadway.

Underground coalmine tunnels are very long and narrow, and some tunnels are approximately thousands of kilometers in length but only several meters in width. For example, in the mines of the Nanyang Coal Industry Co., Ltd., Hengyang, China, the main haulage roadway is approximately 12,000 m long, and most return airways have lengths of more than 1000 m (Figure 1 shows part of the haulage roadway). Coalmining tunnels can be categorized according to their function: haulage roadways, main return airways, development roadways, and preparation roadways. Haulage roadways are used for transportation and act as the main ventilation source for the entire mine, and their dimensions and circumstances are superior to those of other tunnel types; therefore, WSNs can be used. This paper therefore focuses on the haulage roadway as the research objective. If a tunnel has only one sink, information collected by the sensor nodes is transmitted from inside the tunnel to the sink at the tunnel entrance; the sink then transmits all of the data to the control center on the ground. However, the WSN used in underground coalmine tunnels is different from that of normal WSN in several ways. First, tunnels contain various types of complicated elements, such as methane, dust, and explosive gases so it is very difficult to transfer data reliably. In addition, it is impossible to change the batteries of sensor nodes in tunnels; therefore, the development of prolonged network lifetime is a primary research focus. Second, the coalmine tunnel is usually more than several kilometers long; therefore, if only one sink is located at the entrance of the tunnel, it cannot be guaranteed that the cluster head nodes, which are located far from the sink, will be able to transmit data to the sink. Furthermore, these cluster head nodes have large energy consumption and thus require a large amount of energy, which decreases the network’s lifetime. Third, in relation to the cluster head selection, the principle of rotation needs to be adopted to avoid premature damage to the cluster heads. In addition, the distance between the nodes and the sink needs to be considered with respect to residual energy to ensure that the sensor nodes that are located near the sink (and which have higher energy) can become the cluster heads.

Existing studies on the use of WSNs in coalmines have focused on the single-sink network structure. However, there is a major problem inherent in this type of structure: the sensor nodes, which are located far from the single-sink, are unable to promptly transmit data. To solve this problem, this paper proposes the use of a multisink WSN structure and presents a new routing protocol for multisink WSNs that is based on transmission power control and energy balance (PCEB-MS). The basic premise is as follows: a number of sink nodes are placed in the middle of the coalmine tunnel at random intervals (i.e., not more than the maximum communication radius of the sink), and the transmission power between the sinks is controlled to ensure network coverage. In the coverage of each sink, an individual region, which includes the cluster head and universal nodes, optimizes the selection mechanism of the candidate cluster head using a non-uniform clustering idea to ensure that the sensor node that has the largest residual energy becomes the priority candidate cluster head. To enable this step, it is necessary to calculate the non-uniform competition radius and the competition method required by the candidate cluster heads, by considering the distance from the residual energy and the cluster head to the sink. Selection of the cluster head is necessary to enable the energy consumption balance.

The main innovative points of this paper include the following:

(1) The use of multisink WSNs in a coalmine tunnel to enable transmission of data to the sink in a rapid and timely manner.
(2) The provision of a new routing protocol in a coalmine tunnel based on a multisink WSN structure.
(3) The new algorithm has good network performance, including good connectivity, power efficiency, and delay.
(4) By targeting underground coalmine tunnels with multisink WSNs, each sensor node can transmit data to the sink in either one hop or two hops.

**Figure 2:**
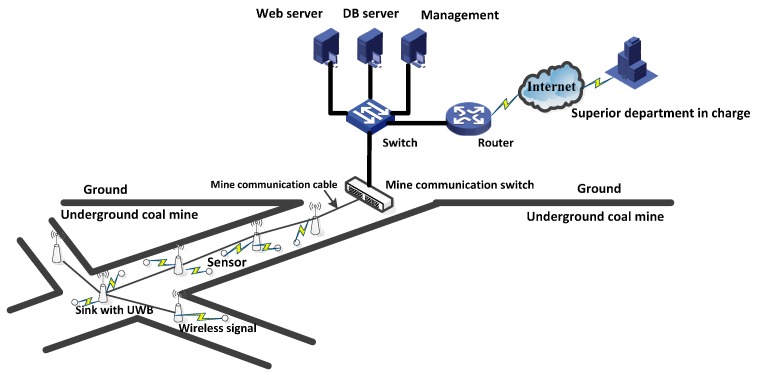
Architecture of multisink WSNs.

Figure 2 shows the architecture of the multisink WSN, which includes more than one sink, many sensor nodes, and one mine communication switch in the underground coalmine. The sink and sensor nodes obtain location information with UWB technology [3,4], the sink nodes connect each other to the mine communication cable, and the sensor node transfers data in one hop or two hops to the sink. All of the data are sent to the mine communication switch and are then transferred to the switch, certain types of servers, and routers on the ground. Finally, the relevant department obtains production information in real time.

This paper is organized as follows: Section 2 describes related studies. Section 3 explains the network and transmission power control model used in this paper. Section 4 presents the detailed design and introduces the PCEB-MS algorithm. Section 5 analyzes the parameters in the PCEB-MS algorithm. Moreover, Section 6 presents the simulation experiment for this study. The final section provides the conclusion and future research directions.

## 2. Related Studies

Most of the existing clustering routing protocols for WSNs, including LEACH (Low Energy Adaptive Clustering Hierarchy) [5], TEEN (Threshold sensitive Energy Efficient sensor Network) [6], and PEGASIS (Power-Efficient GAthering in Sensor Information Systems) [7], adopt a single-sink network structure with a cluster head node and one hop direction communication to the sink. This cluster routing protocol is suitable for large-scale WSNs [8,9]. However, when the protocol applied to a large-scale coalmine network that has a long strip structure, the cluster head that is located far from the sink needs to operate at a large power status, which increases the energy consumption and also causes problems of interference with data transmission over long distances. The concept of using a clustering and multisink network structure can thus be used to solve such issues in a large-scale long-strip network within a coalmine.

Many of the existing studies on multisink WSNs have aimed to prolong network lifetime by studying the optimal number and best deployment location of the sink in the network. In [10], the k-means iterative clustering technique is used to obtain the optimal sink number and to confirm the best location of each sink. A new method based on gene expression for solving the problem of the best deployment location of a sink in a multisink network (GEP-MSN) was suggested by [11]. Recently, Kim et al. [12] introduced a multisink deployment optimization algorithm based on the greedy algorithm, which minimizes data delay to effectively support real-time application. Similarly, Das et al. [13] presented the Centroid of the Nodes in a Partition (CNP) and Candidate Location with Minimum Hop algorithms to optimize sink deployment; out of the two ideas the assumption and simulation inherent in CNP has a greater similarity to that presented in our study.

Another hotspot of multisink WSN research focuses on how to achieve load balance in the network, thus focusing on how to reduce packet loss caused by congestion and collision in the transmission process. In this respect, Erman et al. [14] suggested a network load balancing protocol based on cross-layer partitioning that combines the load balancing and clustering techniques in the whole network, thereby enabling the multisink network to effectively transmit data by constructing the best routing tree based on the local metric. A routing algorithm to achieve load balance between multisinks was further introduced by [15], where each data packet randomly selects only one sink as its target node. In this, the quotient of the least number of hops reaching the target node, divided by the residual energy of the neighbor nodes, is used as the positive factor to determine the route of the next hop. In addition, a gradient-based load balancing algorithm was created by [16], which allows dynamic selection of data-forwarding nodes, based on the residual energy of the next hop campaign node as the forwarding data. The algorithm presented in [17] was then based on this gradient, and by regarding the accumulated load in the routing pathway as the gradient the algorithm is used to solve the inability of the sensor node to avoid selecting the route with the heaviest load. The multisink routing algorithm, based on ant colony optimization, is also similar to the algorithm based on the gradient. For example, the algorithm presented in [18] selects the next hop node according to the pheromone level stored in the routing table. Moreover, in [19], the data are transferred to the multisink by means of the pheromone level. Other multisink routing protocols include research based on fuzzy theory by [20], research based on self-adaptive learning by [21], and research based on partition coverage by [22].

Most algorithms in existing studies that relate to wireless sensor routing focus on energy consumption. In this respect, the use of the competitive mechanism of the cluster head and unequal competitive radius within the long-strip structure of the coalmine area was optimized by [1]. In [23], the LEBUC (Liner Energy-Balanced Uneven Cluster) algorithm was proposed to optimize the competitive mechanism of the cluster head by considering the distance between the sensor node and the sink, the residual energy of the sensor node, and the sensor node density in coalmine. Moving objects in a coalmine tunnel have very-strong temporal and spatial correlations in path selection, and these are considered in [24], where a routing algorithm is proposed on the basis of Bayesian decision by analyzing the probability of path selection by the moving objects in the tunnel. Other partition strategies, including energy balance and cluster head selection according to the tunnel characteristics of the coalmine, were introduced in [25,26,27]. Some novel algorithm is proposed in [28,29]: in [28] a model for underground mines is generated by adopting a performance-based approach, while, in [29] a green MAC algorithm is proposed for smart home sensor networks. These strategies effectively extend the network lifetime and improve network performance.

However, many of the aforementioned studies on multisink routing protocols have not been conducted in accordance with coalmine tunnel characteristics and are therefore not suitable for use in underground coalmine tunnels. Most of the multisink studies as represented by [10,11,12] focused on prolonging the network lifetime by optimizing the number and deployment location of sinks in the network. However, the sensor nodes are usually deployed in a place where it is very difficult to conduct manual maintenance within the coalmine tunnel. Some studies [14,15,16,17,18,19,20,21,22] have proposed methods relating to load balance and effectiveness of energy consumption with the aim of avoiding packet loss caused by congestion and collision in the data transmission process. However, these studies are not directly related to the special structure of the coalmine tunnel and therefore cannot be applied here. In addition, although other studies [1,23,24,25] have investigated energy consumption in accordance with the special coalmine tunnel structure, such studies have unfortunately been based on a single-sink structure, which has evident application limitations.

It is thus evident that the previous studies are not applicable for use in underground coalmine tunnels, because they are either based on the single-sink network structure or they do not consider the special shape of underground tunnels. However, because the CNP algorithm is based on a no-grid-cell multisink structure, sensor nodes are deployed randomly in its simulation, and the algorithm consumes less energy and has a longer lifetime, we compare the CNP algorithm with the algorithm presented in our study. Furthermore, the CNP algorithm uses the one hop rule and has a similar calculation method to our algorithm. 

Our paper focuses on transmission power control and combines the multisink network structure with clustering routing technology to propose a new routing protocol known as PCEB-MS for underground coalmine tunnels, focusing particularly on the haulage roadway. This protocol obtains an optimal communication radius by negotiating the sink transmission power control to guarantee the coverage area of a network. The unequal clustering algorithm is also used to ensure the energy balance of the network to obtain the best connectivity, prolong the network lifetime, and rapidly transmit monitored data.

## 3. Network and Power Control Model

### 3.1. Network Model

The WSN investigated in this study is designed to be mainly used in certain coalmine tunnels that are, in particular, within the haulage roadway and that have a long strip shape. As the WSN has a length that is much greater than its width, several sinks are needed within the chain. In addition, the communication radius and transmission power between all the sink nodes are negotiated to ensure WSN coverage in the coalmine tunnel. In relation to the coverage of each sink, unequal clustering is established in individual regions to optimize network topology, reduce the overall energy consumption of the network and clustering interference, and prolong the network lifetime with the aim of ensuring the best connectivity.

The PCEB-MS protocol used in the coalmine tunnel, which is discussed in this paper, sets a sink at random intervals in the center of the link, which is not greater than the maximum communication radius of the sink. Each sink has its own location by means of the UWB technology, and it broadcasts a power control message (PCM) within its communication radius. Sink nodes communicate with one another using a special mine communication cable buried in the ground. If we assume that two sink nodes are able to communicate in the initial stage, each sink then receives monitoring information from a sensor node within a certain region, which ensures the PCM exchange between both sinks. 

In this paper, we suppose that the number of sensor nodes in the network is N, and these are distributed in a long-strip region measuring *L* × *W* with *L* >> *W*. As soon as the sink and sensor node are deployed, the location is fixed and it no longer changes, but the transmission power of the sensor nodes and the sink is controllable. The distance between nodes and the receiving power are calculated on the basis of the intensity of the receiving signal. The receiving power is more than the minimum power threshold of the normal receiving data *PR*_min_. The sensor nodes are isomorphic with the same initial energy, and they also have data fusion function and self-sensing of residual energy. Furthermore, there is unrestricted sink energy.

### 3.2. Power Control Model

The working power of each sink is negotiated to ensure network coverage and connectivity. 

Generally, the propagation range of the electric wave in the tunnel is divided into two areas: the propagation near zone and the propagation far zone. When the distance between the transponders is less than the maximum communication radius of the sink, it belongs to the near zone. In this area, the guided communication is not established, radio waves are mainly conducted in multi-mode propagation mode, and most of them belong to high-order mode. This propagation is similar to the free space model, so we use the propagation model. Furthermore, the process of confirming the appropriate transmission power in the power control mechanism was described by [30,31,32]. Thus, we use the propagation model and base on the following assumptions: First, the physical layer can transmit a frame at one of the discrete power levels notified by the MAC layer. Second, the physical layer notifies the MAC layer about RSSI of a received frame.

The receiving power of the node is obtained as follows based on the Friis formula of propagation loss of the electric wave in free space:

(1) $$P_{r}=P_{t}g_{t}g_{r}{\left(\frac{\lambda }{4\pi d}\right)}^{n},$$

where *P_r_* is the receiving power of the node; *P_t_* is the transmission power of the node; *g_t_* and *g_r_* are the gains of the transmitting and receiving antennas, respectively; *d* is the distance between the transmitting and receiving nodes; and *λ* is the carrier wavelength decided by the carrier frequency. *λ* is usually 0.1 in the commonly used 2.4-GHz wireless sensor; *n* refers to the channel fading coefficient, which is usually 2; and *g_t_*, *g_r_*, and *λ* are the parameters of the sensor network. The values of these parameters are determined for a confirmed network and are expressed by *ϕ* as follows:

(2) $$\varphi =g_{t}g_{r}{\left(\frac{\lambda }{4\pi }\right)}^{2}.$$

The formula of the node receiving power is then rewritten as

(3) $$P_{r}=P_{t}\frac{\varphi }{d^{2}}.$$

Furthermore, based on the preceding formula, the transmission power from the sink to the node in its coverage is written as

(4) $$P_{t}=\frac{P_{r}d^{2}}{\varphi }.$$

The receiving power is calculated on the basis of the intensity of the receiving signal, as shown by [32]. The optimal transmission power is calculated using Equation (4), where *d* is equal to the best communication radius of the sink. Therefore, the control problem of the transmission power is converted to the control problem of the communication radius.

## 4. Design of PCEB-MS Protocol

The PCEM-MS protocol involves two parts: the distributed power control algorithm (DPCA), based on the multisink DPCA, and the unequal clustering and energy-balanced algorithm located inside the sink (UCEBA).

The DPCA based on the multisink is mainly used to adjust the power of the adjacent sink and ensure the overall multisink coverage. The UCEBA achieves energy balance and prolongs network lifetime by considering the long-strip structure of the coalmine tunnel.

### 4.1. DPCA Algorithm

According to the power control model, the control problem of the transmission power is converted to the control problem of the communication radius, and the following theorem is obtained.

**Theorem** **1.** Two sinks (S1 and S2) are deployed in a rectangular area, P, with a size of a × b, and in the central line of the rectangular area. It is necessary for the wireless range circling the two sinks to intersect outside of the area P to guarantee sink coverage in the network. In addition, the rectangular top point corresponding to the sink location needs to be within the wireless range of the sink. Here we use R_S1_ to represent the wireless range of S1 and R_S2_ to represent the wireless range of S2.

**Figure 3:**
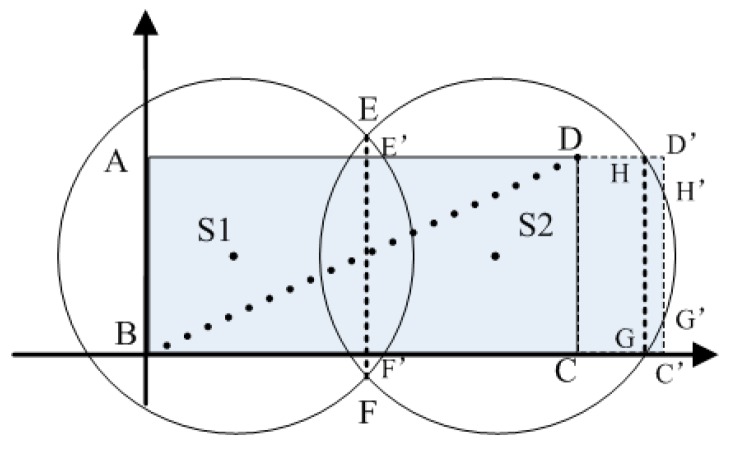
WSN with two sinks.

**Proof.**  Two sinks are distributed in the *ABCD* rectangle area with a size of *a* × *b* (Figure 3). The two circling sink wireless ranges intersect at *E* and *F*. The following relation thus needs to be met to ensure network coverage:

(5) (AEFB∪EDCF)⊇ABCD.

Accordingly, *EF* is the connecting line between the two intersecting points of two circles. This line intersects with line *AD* of the rectangle at *E*′. *F*′ intersects with line *BC* of the rectangle. Therefore, $EF\geq E^{\prime }F^{\prime }$ needs to be met to satisfy Equation (5), and it is necessary for the two circling wireless ranges of the sink to intersect outside region P.

If we suppose that the rectangular vertices corresponding to sink *S2* are not inside its wireless range (Figure 2), the rectangular vertices are then extended to *D*′ and *C*′, and rectangle *ABC*′*D*′ intersects with the wireless range circle *S2* at *H*, *H*′, *G*, and *G*′. However, the range covered by the circling wireless range of the two sinks does not cover the entire rectangular *ABC*′*D*′ area,

(6) $$(AEFB\cup EHGF\cup HH^{\prime }GG^{\prime })\subset ABC^{\prime }D^{\prime }.$$

As Equation (6) does not meet the requirements of Equation (5), the rectangular vertices corresponding to the sink need to be located in the wireless range of the sink to ensure network coverage. ☐

According to Theorem 1, the wireless ranges of *R_S_*_1_ and *R_S_*_2_ should meet the following formula,

(7) $$\left\{ \begin{array}{l} {\left(x_{s1}-x\right)}^{2}+{\left(y_{s1}-y\right)}^{2}=R_{s1}^{2}\\ {\left(x_{s2}-x\right)}^{2}+{\left(y_{s2}-y\right)}^{2}=R_{s2}^{2} \end{array}\right.,$$

where (*x*, *y*) refers to any sensor node in the sink communication range, and (*x_s_*_1_, *y_s_*_1_) and (*x_s_*_2_, *y_s_*_2_) refer to the coordinates of the two sink nods. The transmission power of each sink is minimized to ensure network coverage. Function *f* is defined as follows:

(8) $$\begin{array}{l} f=\text{min}\sum_{i=1}^{2}R_{si}^{2}\\ s.t.\\ {\left(x_{s1}-x_{A}\right)}^{2}+{\left(y_{s1}-y_{A}\right)}^{2}\leq R_{s1}^{2}\\ {\left(x_{s2}-x_{C}\right)}^{2}+{\left(y_{s2}-y_{C}\right)}^{2}\leq R_{s2}^{2}\\ x_{E}>0,x_{F}<a\\ y_{E}\geq b,y_{F}\leq 0 \end{array},$$

Here, the two sinks are taken as examples, but the remaining sinks can be deduced using the same method.

**Figure 4:**
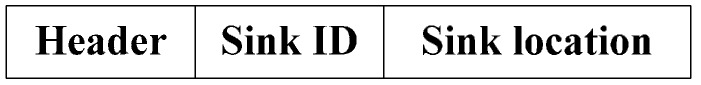
Frame format of a handshake packet in the distributed power control algorithm (DPCA).

Handshake data packet needs to be defined to ensure communication between the sink nodes for the DPCA algorithm. This handshake packet includes three data domains (Figure 4), namely, the packet header, sink ID, and sink location information. Among these domains, the sink ID in ascending order is unique in the network. At network initialization, each sink is able to obtain information relating to its adjacent sink by receiving a handshake packet.

*k* sink nodes are deployed in the network with a size of *L* × *W*, and the coordinates are represented by (*x_si_*, *y_si_*), where 1 ≤ *i* ≤ *k*. *P*(*X*, *Y*) is set as a rectangular area, where *X* is the length of the rectangle and *Y* is the width of the rectangle, and *X* > *Y*. *Cir*(*x_si_*, *y_si_*, *r_si_*) is a circular region with (*x_si_*, *y_si_*) as the center and *r_si_* as the radius. The following then needs to be met to ensure 100% network coverage,

(9) $$P\left(X,Y\right)\subset Cir\left(x_{1},y_{1},r_{1}\right)\cup Cir\left(x_{2},y_{2},r_{2}\right)\cup \ldots \cup Cir\left(x_{k},y_{k},r_{k}\right).$$

It is necessary for each sink to have appropriate transmission power after negotiation to ensure that each sink reaches 100% network coverage after transmission power control. Equation (4) indicates that the transmission power is calculated after obtaining the communication radius of each sink. Therefore, calculation of transmission power is converted to the solution of the communication radius.

In this respect, each sink firstly obtains its own coordinate information using the UWB technology. A handshake packet and a PCM message are then broadcasted to the neighboring sink. The distance between the sink with the minimum sink ID and the corresponding top of the rectangular area are regarded as the wireless range. 

There are four intersection points between the wireless range circle and the rectangular boundary of this sink (S1 in Figure 3). The other sinks also have four intersection points, if an appropriate wireless range is selected (S2 in Figure 3). After the wireless range is determined, each sink then sends a sink ID message to all the sensor nodes in its coverage. Moreover, this sink ID message is used in the clustering algorithm. For the overlapping region with two sinks, the sensor node will obtain two sink IDs, and these sensor nodes then select the closest sink and transmit the data to the sink directly.

In this study, it is considered that the sink coverage is guaranteed for the entire network once the union of the cutting line contains four lines of the entire rectangle, and after the circle (with each sink as the center of the circle) and the corresponding wireless range (as the radius) intersect with the rectangle’s lines. The wireless range, *r_i_*, starting from the second sink, i∈k, must then meet the following relation,

(10) $$X\subset \left(2x_{1}\right)\cup \sqrt{r_{2}^{2}-{\left(x_{2}-2x_{1}\right)}^{2}}\cup \ldots \cup \sqrt{r_{i}^{2}-{\left(x_{i}-2x_{i-1}\right)}^{2}}.$$

**Figure 5:**
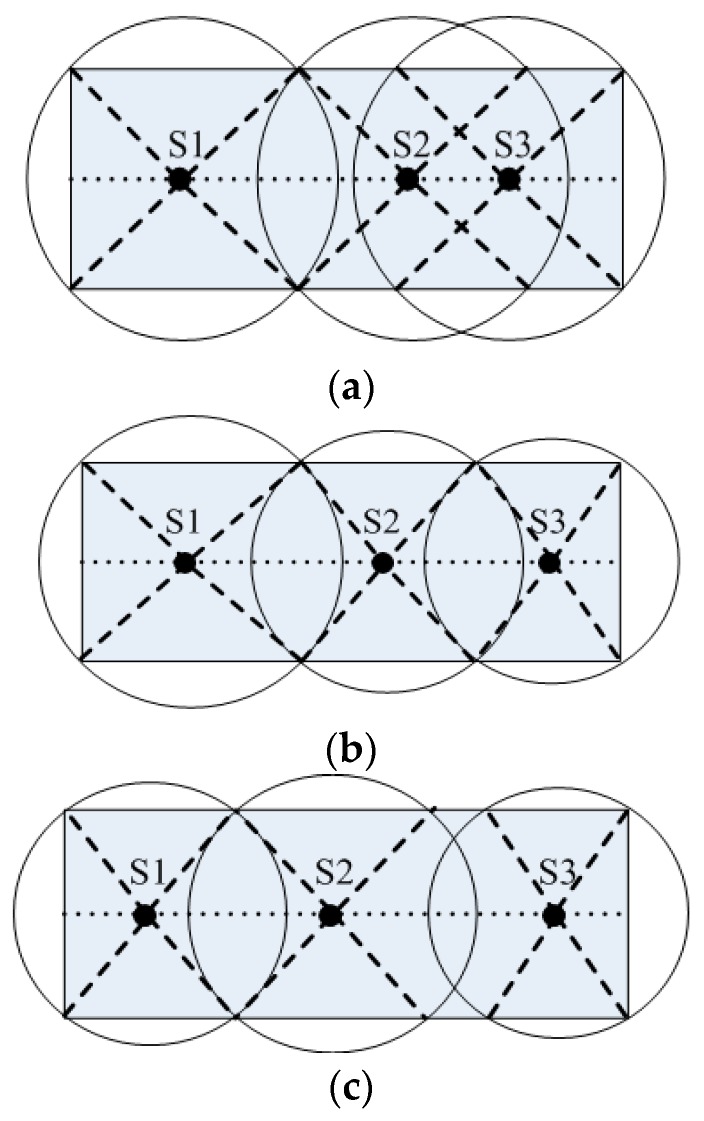
Selection of wireless ranges by three sinks in the network: (**a**) extremely large; (**b**) suitable; and (**c**) extremely small.

Based on this theory (Figure 5), the following three selections of the sink for the wireless range are found, if three sinks exist in the network: (a) extremely large; (b) suitable; and (c) extremely small. Calculations are then required for each sink to obtain the most ideal wireless range, and then each sink can select the proper transmission power by automatic adjustment.

**Figure 6:**
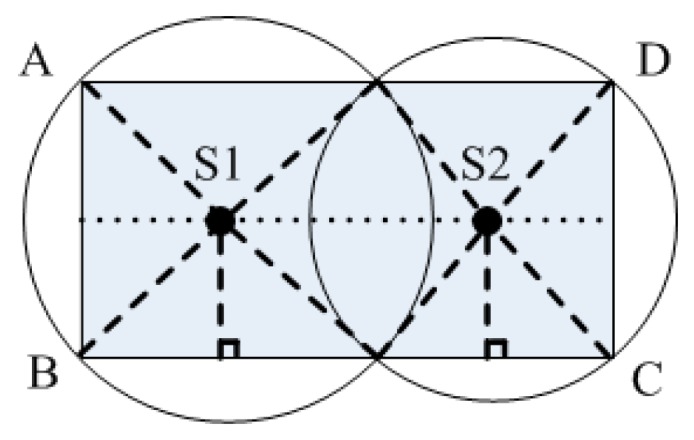
Calculation process for determining the sink wireless range.

Figure 6 introduces the calculation process for determining the wireless range. Two sinks are deployed in S1 and S2: the coordinates of S1 are (*x_s_*_1_, *y_s_*_1_) and those of S2 are (*x_s_*_2_, *y_s_*_2_). S1 is the first sink with sink ID 1. This sink regards |S1B| as its wireless range (i.e., *R_S_*_1_). S2 therefore needs to calculate its wireless range (*R_S_*_2_) based on R_S1_,

(11) $$R_{S2}=\sqrt{{\left(\left(x_{S2}-x_{S1}\right)-\sqrt{R_{S1}^{2}-y_{S1}^{2}}\right)}^{2}+y_{S2}^{2}}.$$

The wireless range *R_Si_* of each sink is then represented by

(12) $$\left\{ \begin{array}{cc} R_{Si}=\sqrt{{x_{Si}}^{2}+y_{Si}^{2}} & i=1\\ R_{Si}=\sqrt{{\left(\left(x_{Si}-x_{S(i-1)}\right)-\sqrt{R_{S(i-1)}^{2}-y_{S(i-1)}^{2}}\right)}^{2}+y_{Si}^{2}} & 2\leq i\leq k \end{array}\right..$$

The UCEBA proposed in this paper is used for clustering after the transmission power of each sink has been adjusted. After clustering has been completed, the sensor nodes in each cluster initiate a one hop communication with the cluster head in the same cluster. Finally, the cluster head sends data to the sink using the one hop communication, thereby ensuring that the data are rapidly and stably transmitted to the sink from the sensor node.

**Figure 7:**
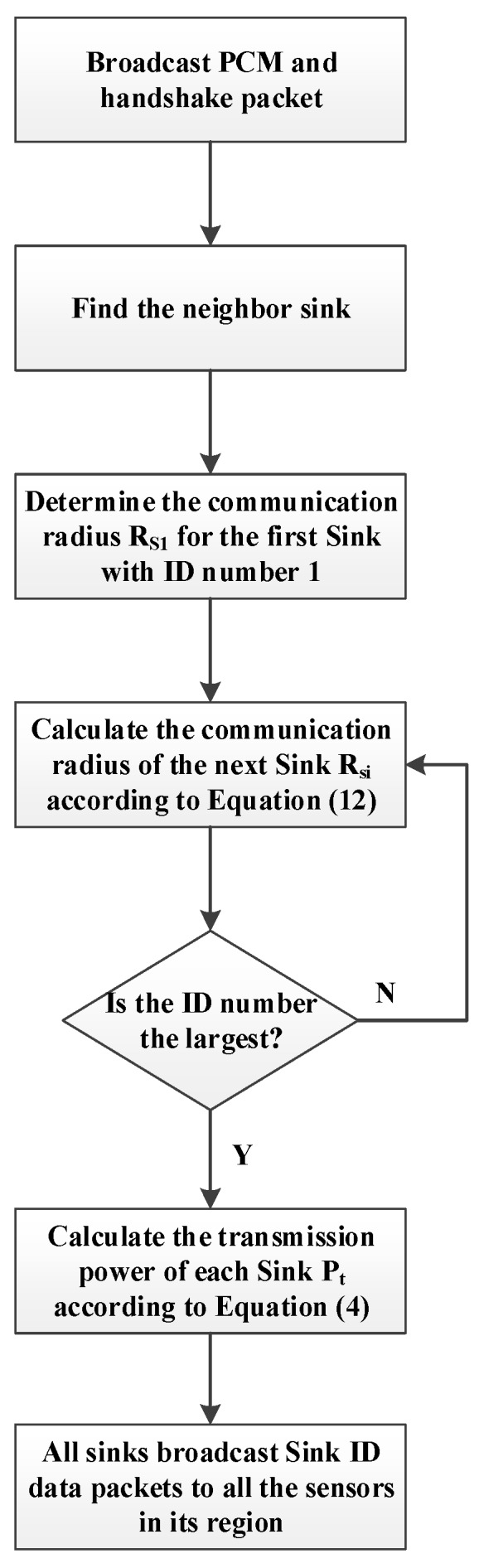
Procedure of DPCA algorithm.

Transmission power maintenance is a process that involves adjusting the sink’s own transmission power in real time according to the actual working environment. After a route becomes invalid, the sensor node begins an alternate route or routing mechanism, and this causes each sink to rearrange its transmission power. The wireless range of the sink is estimated by the DPCA algorithm on the basis of the link between the local sinks. Therefore, its power maintenance is realized through the periodic exchange and broadcasting of the PCM. Figure 7 describes the DPCA algorithm.

### 4.2. UCEBA Algorithm

**Figure 8:**
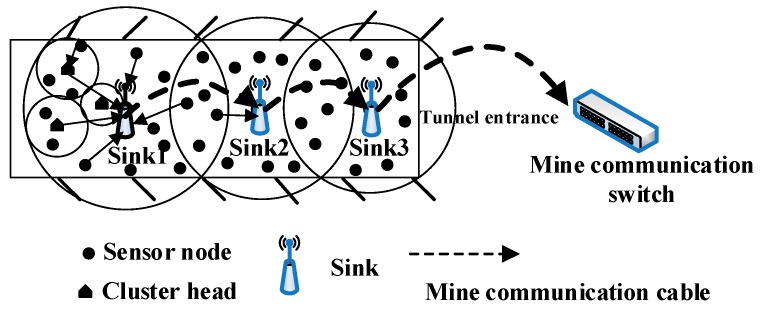
Unequal clustering mechanism.

The UCEBA algorithm is mainly used in the selection of the cluster head, calculation of the unequal campaign radius, and generation of the final cluster head. Each sensor node joins the cluster in one hop. For the overlapping region with two sinks, the sensor node will obtain a two sink ID; these sensor nodes select the closer sink and transmit the data to that sink directly. To guarantee the energy balance and to prolong the network lifetime, the cluster that is furthest from the sink has a larger radius and more cluster members than the cluster that is closer to the sink. Figure 8 shows the specific clustering mechanism involved. Accordingly, the cluster near sink 1 has a small radius, whereas that which is furthest from sink 1 has a large radius. The communication process is described as follows. First, the sinks are set in the middle of the coalmine tunnel at random intervals (i.e., not more than the maximum communication radius of each sink). Second, the transmission power between the sinks is controlled to ensure network coverage dynamically based on the DPCA algorithm. Third, the clusters are built within the coverage of each sink as individual regions using the UCEBA algorithm; the transmission power of the sensor nodes can then be controlled to guarantee minimum energy consumption. Finally, the sensor nodes use one hop or two hops to transmit data to the sink directly or via the cluster head.

#### 4.2.1. Selection of the Candidate Cluster Head

After power adjustment, the obtained coverage areas, all the sinks that broadcast the sink ID data packet in their coverage areas, and the sensor nodes that receive the same sink ID are considered to be in the same region. Clustering is then conducted in this region. Some of the sensor nodes located in the overlapping region will obtain a two sink ID; these sensor nodes will then transmit data directly to the closer sink without needing to join a cluster.

In selecting the candidate cluster head, a threshold value, *T*, is firstly preset. A *u*_0_ value is then randomly generated in each sensor node in [0, 1]. If this value is smaller than *T*, then the sensor node becomes the candidate cluster head. The calculation formula of *u*_0_ is shown as follows:

(13) $$u_{0}=u\frac{E_{0}}{E_{cu\left(i\right)}},$$

where *E*_0_ refers to the initial energy of the sensor node, and *E_cu_*_(*i*)_ refers to the residual energy of *i* sensor node. *u*_0_ is then calculated using Equation (13) after generating *u* randomly. A sensor node is selected as the candidate cluster head if *u*_0_ ≤ *T*. Moreover, a sensor node with larger residual energy has a smaller *u*_0_ after calculation and is thus more likely to become the candidate cluster head. A sensor node with *u*_0_ > *T* become a non-candidate cluster head and it then enters a sleeping state.

#### 4.2.2. Calculation of Unequal Radius

It is necessary to have an unequal competitive radius to control the cluster scale and to determine the final cluster head out of all candidate cluster heads. A certain relation clearly exists between the unequal radius and the residual energy of the node; the radius becomes larger if the residual energy is higher and the scale is larger. In contrast, the radius becomes smaller if the residual energy is lower, which effectively prolongs the network lifetime. The competitive formula proposed in this paper is presented as follows:

(14) $$R_{ra}=\left[1-\omega_{1}\left(1-\frac{E_{cu}}{E_{0}}\right)-\omega_{2}\left(1-\frac{d_{toSK}}{d_{\text{max}}}\right)\right]R_{\text{max}}$$

Accordingly, *ω*_1_ and *ω*_2_ refer to the adjustment coefficient in the 0–1 range. *E_cu_* refers to the residual energy of the existing candidate cluster head node, *E*_0_ refers to the initial energy of the candidate cluster node, *d_toSK_* refers to the distance between this candidate cluster head node and the sink, *d*_max_ refers to the maximum distance between all the candidate cluster heads and sink, and *R*_max_ refers to the maximum cluster radius. Clearly, exceeding *R_si_* is impossible for the maximum cluster radius, *R*_max_. Therefore, this study considers that *R*_max_ ≤ *R_si_*.

An analysis of Equation (14) shows that if the residual energy of the candidate cluster head is less than others and has a smaller competitive radius, then the premature death of the cluster head is avoided. In addition, if the distance between the candidate cluster head and sink is shorter, then the competitive radius is also smaller than others, thereby avoiding the premature death of the cluster head caused by heavy forwarding of data. Therefore, a competitive radius is used to control the scale of the cluster head and to ultimately achieve an energy balance.

#### 4.2.3. Selection of Final Cluster Head and Cluster Formation

The final cluster head is generated using a certain competitive strategy. The sensor nodes are unable to supply energy for the coalmine tunnel once they are deployed; therefore, it is necessary to prolong the lifetime of the sensor nodes that are located far from the sink and have low energy. That is to say, the sensor which is closer to the sink node and higher in energy is preferred as the cluster head, thus prolonging the network lifetime. 

First, each sensor node for the UCEBA saves a list of neighbor nodes. Table 1 presents information relating to neighbor sensor nodes. All sensor nodes receiving the same sink ID are located in the same region. Some of the sensor nodes in the overlapping region will obtain a two sink ID, and these sensor nodes transmit data directly to the closer sink and do not need to join any cluster. Table 1 shows that the list of neighbor nodes comprises the candidate cluster head ID, status, residual energy, and distance to the sink. Each candidate cluster head broadcasts with a competitive radius, *R_ra_*, as it transmits its competitive information COMPETE_MSG. All candidate cluster heads in radius *R_ra_* of a candidate cluster head, *V_i_*, are regarded as neighbor nodes of this candidate cluster head. The neighbor node of the candidate cluster head *V_i_* is recorded as *V_i_*.Neitab.

*V*_i_.Neitab = {*V_j_*|*V_j_* refers to the candidate cluster head and range (*V_i_*, *V_j_*) < max(*V_i_*.*R_ra_*, *V_j_*.*R_ra_*)},

where range (*V_i_*, *V_j_*) denotes the distance between nodes *V_i_* and *V_j_*.

Each candidate cluster head calculates the average residual energy, *E_a_*, of all the sensor nodes in its competitive radius and the average distance, *D_a_*, to the sink according to the list of neighbor nodes.

(15) $$E_{a}=\frac{\sum_{j=1}^{m}V_{j}.E_{cu}}{m},$$

(16) $$D_{a}=\frac{\sum_{j=1}^{m}V_{j}.d_{toSK}}{m}.$$

Accordingly, *V_j_* (1 ≤ *i* ≤ *m*, *i* ≠ *j*) refers to the neighbor sensor node in a radius, *R_ra_*, of the candidate cluster head *V_i_*, and *m* refers to the number of neighboring sensor nodes.

Each candidate cluster head then starts timer, *t*, according to the following equation,

(17) $$t=k\times t_{0}\times \frac{E_{a}}{E_{cu}}\times \frac{d_{toSK}}{D_{a}},$$

where *k* refers to the real number evenly distributed in [0, 1] at random, and *t*_0_ refers to the competitive duration of the candidate cluster head agreed in advance. The candidate cluster head node is successfully competitive if it does not receive the message on the competition victory of the neighbor candidate cluster head node SUCCESS_MSG before time, *t*. The message SUCCESS_MSG is then sent to the other neighbor candidate cluster nodes. Otherwise, the candidate cluster head is defeated and quits the competition. The aforementioned formula and analysis show that all the sensor nodes in the competitive radius have relatively high residual energy. Moreover, there is a higher probability that a sensor node that is located relatively near the sink will become the final cluster head, and will ultimately and effectively prolong the lifetime of the candidate cluster head nodes located far from the sink that have lower energy.

The sensor nodes that do not participate in the competition are woken from their sleeping state after the final cluster head has been selected through competition. The final cluster head broadcasts the competition victory message, CH_ADV_MSG, using *R_ra_* as the radius. The ordinary sensor nodes select the cluster head that has the minimum communication cost from many neighboring cluster heads, namely, the cluster head with the strongest signal-receiving intensity, to send the message JOIN_CLUSTER_MSG, and to note the cluster head. This cluster head finally constructs the TDMA dispatch using the organization method, which is similar to the LEACH protocol, and transmits data by clustering members to the cluster head. Algorithm 1 shows the process of a sensor node, *V_i_*, participating in the cluster head competition and finally forming a cluster.
**Algorithm 1:** UCEBA Algorithm: unequal cluster algorithmInput: initial energy of the *E*_0_ node; residual energy of *i* of the *E_cu_*_(*i*)_ node; distance *d_toSK_* between the candidate cluster head and the sink; the maximum distance *R*_max_ between the candidate cluster head and the sink; duration of the competition of the candidate cluster head agreed in advance *t*_0_Output: cluster head, unequal radius *R_ra_*01:Sensor nodes with the same sink ID form a region.A sensor node in the overlapping region obtains two sink IDs and the distance between it and the two sinks is calculated. The sensor node then transmits data to the closer sink and does not need to join a cluster.02:Other sensor nodes that only have one sink ID are used to calculate *u*_0_ according to Equation (13), and are then compared with the threshold value, *T*; those with a value less than *T* become the candidate cluster head (if not, they become an ordinary sensor node). The unequal radius, *R_ra_* is then calculated according to Equation (14).03:Each candidate cluster head broadcasts its competitive message, COMPETE_MSG, using a competitive radius *R_ra_*.04:Each candidate cluster head starts timer, *t*, according to Equation (17).05:The candidate cluster head is successfully competitive if each candidate cluster head does not receive the message SUCCESS_MSG from the neighbor candidate cluster head before *t* time is shown in its timer. This indicates that the candidate cluster head has been successfully competitive. After it becomes the cluster head, it then proceeds to 07; otherwise it proceeds to 06.06:The candidate cluster head defects and quits the competition, thereby becoming an ordinary sensor node.07:The candidate cluster head is successfully competitive, becomes a cluster head node, and sends a competition victory message, SUCCESS_MSG, to all neighboring candidate cluster head nodes.08:The cluster head node broadcasts the competition victory message, CH_ADV_MSG, to the ordinary sensor nodes, using *R_ra_* as the radius.09:The ordinary sensor node sends message, JOIN_CLUSTER_MSG, to notify the cluster head that it will become a cluster member node for sending data to the cluster head.10:End


## 5. Analysis of PCEB-MS

The aim of this section is to analyze and explain the involvement of parameters *T*, *ω*_1_, *ω*_2_, and *R*_max_ in the PCEB-MS protocol.

Firstly, the value of *T* directly influences the number of candidate cluster heads. This value cannot be extremely large or small; if the value is too large, the candidate cluster head number will be too many, thus increasing the election expenses, reduce the network lifetime, if the value is too small, cannot reflect the advantages of clustering. Therefore, determining a reasonable value for *T* is very important in the optimization of network energy consumption; in this paper, the value of *T* is set at 0.4.

The values of *ω*_1_ and *ω*_2_ reflect the influence of the residual energy of the sensor node, and of the distance between the sensor node and the sink, on the competition radius of the cluster. Reasonable values optimize the competitive radius and facilitate the energy balance, and the influence on the competitive radius is proportional to the value of *ω*_1_ and *ω*_2_. Through the simulation experiment, the values of *ω*_1_ and *ω*_2_ are confirmed to be 0.6 and 0.2.

The value of *R*_max_ directly influences the unequal competition radius, *R_ra_*. Clustering occurs after the communication radius of all the sink nodes is adjusted, and therefore, *R*_max_ is not larger than *R_Si_*. Experiments in some of the large-scale coalmines show that the maximum transmission radius of *R*_max_ is 75 m [33]; therefore, *R*_max_ is 75 when *R_si_* is more than 75 m in the calculation process. In addition, the value of *R*_max_ is *R_si_* when *R_si_* is less than 75 m.

## 6. Evaluation of Algorithm Performance

This study constructs a number of network simulation environments to verify suitability of the PCEB-MS protocol for use in the long-strip structure of the coalmine tunnel. The simulation environment is also used to conduct a comparative analysis of the PCEB-MS protocol and the CNP algorithm, with reference to their connectivity and clustering interference. In addition, the influence of an increasing number of sinks on the network’s performance using the PCEB-MS protocol, is also compared in relation to delay and network lifetime.

The simulation platform in the experiment is constructed with OMNET++4.2 as the simulation tool, using the C++ language in the Windows operating system [33,34]. Simple modules are used for the nodes and sinks in the experiment, which are defined as node.ned and Sink.ned, respectively. Table 2 shows the other parameters.

### 6.1. Power Efficiency

It is extremely difficult (and almost impossible) to change the battery of the sensor nodes in the coalmine tunnel, and thus the power efficiency needs to be optimal to ensure that the network lifetime is prolonged. The power efficiency is defined in this section as follows: the sensor nodes in the network reach the sink nodes through one hop or two hops, and the communication radius of each sink is also controlled to obtain the optimal transmission power, which thus minimizes the total energy consumption used from all the sensor nodes to the sink.

If we suppose that *k* sinks are present in the network, then the total transmission power, *P_sum_*, of the sink nodes in the network is expressed as Equation (18), according to the relationship between the transmission and receiving power and the communication distance described in Equation (4),

(18) $$P_{sum}=\sum_{i=1}^{k}P_{t,i}=\sum_{i=1}^{k}\frac{P_{r,i}d_{i}^{2}}{\varphi }.$$

For the hardware energy consumption of the radio, the energy consumption of the transmitter at the time of transmission includes the wireless electronic device and the power amplifier, and the energy consumption of the receiver at the time of reception is only that of a radio electronic device. 

The first-order radio energy consumption model proposed in [35,36,37,38,39] is used in our study; the model considers both the transmitting energy and receiving energy consumption. Based on this model, the energy consumed by the data transmission of *lbit* to *s* is shown as follows,

(19) $$E_{Tx}\left(l,s\right)=\left\{ \begin{array}{l} lE_{elec}+l\varepsilon_{fs}s^{2},s<s_{0}\\ lE_{elec}+l\varepsilon_{mp}s^{4},s\geq s_{0} \end{array}\right.,$$

where *E_elec_* refers to the energy consumed by the transmitting circuit. The free space model is adopted if the transmission distance is less than the threshold value, *s*_0_; otherwise, the multipath attenuation model is used. The value of *s*_0_ is 75 m according to [33]. *Ε_fs_* and *ε_mp_* refer to the energy required by the two models for power amplification. The energy that the sensor node consumes for receiving *lbit* is calculated as follows,

(20) $$E_{Rx}\left(l\right)=lE_{elec}.$$

In addition, *E_DF_* is used to represent the energy used for fusing the unit bit data in this study.

The sensor nodes need to receive the broadcast packets from the sink and to send data to the cluster head node or sink; therefore, the total energy consumption can be expressed by the following equation,

(21) $$E_{total}=E_{Tx}\left(l,s\right)+E_{Rx}\left(l\right)+lE_{DF}=\left\{ \begin{array}{l} lE_{elec}+l\varepsilon_{fs}s^{2}+lE_{elec}+lE_{DF}+E_{x},s<75\\ lE_{elec}+l\varepsilon_{mp}s^{4}+lE_{elec}+lE_{DF}+E_{x},s\geq 75 \end{array}\right.$$

in which *E_x_* is a fuzzy factor. The actual roadway environment is not fully adapted to the first-order radio energy consumption model; however, as the haulage roadway is a horizontal tunnel for transport and ventilation, its conditions are much better and thus closer to this model. We use the fuzzy factor to make adjustments, with the aim of ensuring the experiment can meet the requirements of the model.

From Equation (21), we can see that if we want to reduce the total energy consumption of the sensor nodes, we need not only to consider the process of transmitting data, but also the process of receiving data. When we use the PCEB-MS algorithm, the sensor nodes only require one hop or two hops to transmit data to the sink, therefore reducing the transmitting energy, and they also receive fewer broadcast messages from the sink, thus effectively reducing the total energy consumption of sensor nodes.

In this experiment, the energy consumption of the parameters are set as *ε_mp_* = 0.0013 pJ/bit/m^4^, *E_elec_* = 50 nJ/bit, *ε_fs_* = 10 pJ/bit/m^2^, and *E_DF_* = 5 nJ/bit/signal, which are the same values as those set in [40,41].

**Figure 9:**
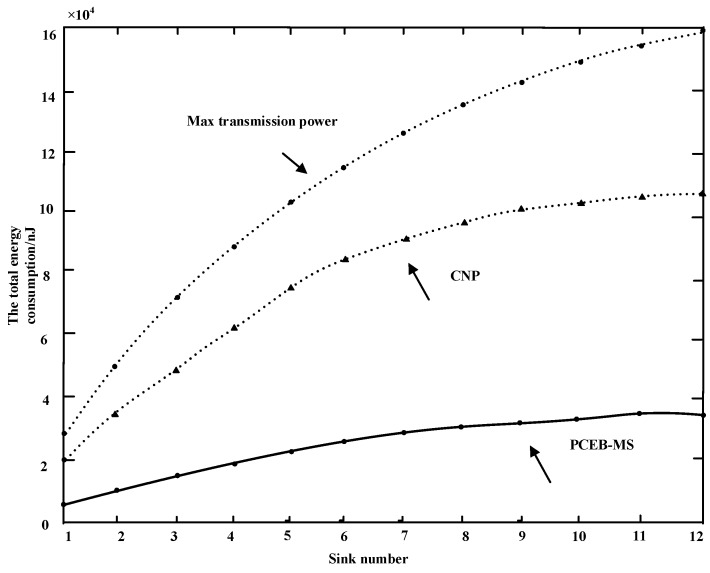
Comparison of total energy consumption of sinks.

According to the relationship between the sink transmission power, transmission radius, and energy consumption presented in [42,43,44,45,46], Figure 9 shows a comparison between the CNP algorithm and the PCEB-MS protocol in relation to maximum power. This shows that the PCEB-MS protocol is superior in saving energy consumption; and thus, with an increase in sink nodes the energy consumption would be lowered. When there are 12 sinks, the total energy consumption is reduced by approximately 70% compared to maximum transmission power, and by approximately 64% with the CNP algorithm. 

### 6.2. Connectivity

After executing the PCEB-MS protocol, the entire network coverage needs to be guaranteed to ensure normal communication between all sensor nodes and sink nodes. This section defines a parameter, known as average connectivity, which refers to the percentage of sensor nodes covered by the sink to all the sensor nodes. With an increase in the number of sinks, connectivity reaches 100% when the number reaches a certain value. All the sensor nodes are covered by the network. In addition, all the experimental results presented are the average values from 1000 repetitions.

**Figure 10:**
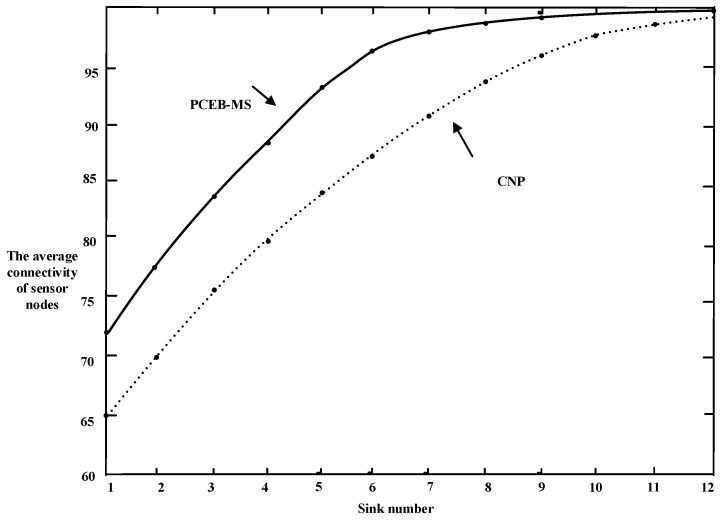
Comparison of average connectivity.

Figure 10 makes a comparison between the PCEB-MS protocol and the CNP algorithm, in terms of the change in the number of sinks and the average connectivity. With an increase in the number of sink nodes in the network, the average connectivity increases correspondingly; when the number of sink nodes reaches 12, the average connectivity is close to 100%. It is evident that the average connectivity obtained using the PCEB-MS protocol is better than that obtained using the CNP algorithm. For example, the initial connectivity derived using the PCEB-MS protocol is 72% when only one sink is present, and that using the CNP algorithm is only 65% for the same number of sinks. When the number of sinks increases to 6, the connectivity obtained using the PCEB-MS protocol is close to 96%, whereas that obtained using the CNP is only 86%. Ultimately, when there are 12 sinks, the average connectivity using the CNP algorithm is slightly lower than that using the PCEB-MS.

### 6.3. Cluster Interference

The most common method used to reduce interference in a cluster is the transmitter-based encoding distribution, which is introduced in [44,45]. In this method, each cluster has independent spread-spectrum encoding, and a unique spread-spectrum code is adopted for the communication between each node in the cluster and the cluster head. The node in the cluster uses this spread-spectrum code to transmit data to the cluster head, and the cluster head node filters the received signal based on the spread-spectrum code.

**Figure 11:**
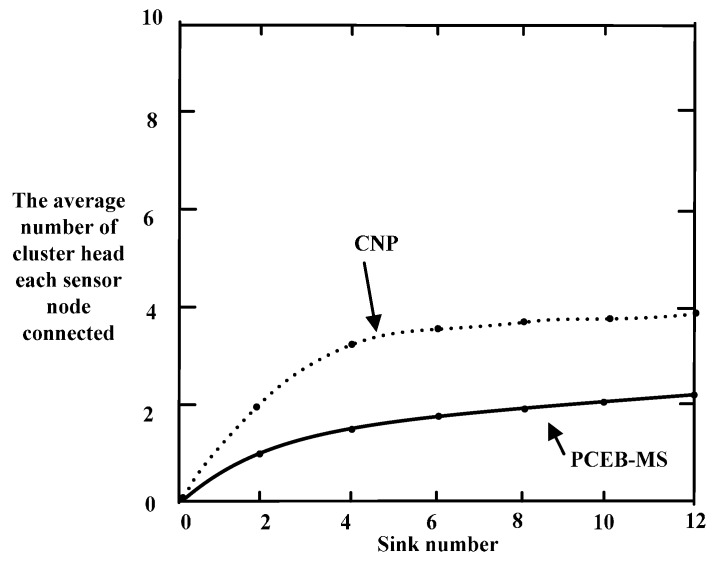
Cluster interface comparison.

In this paper, cluster interference is defined as the number of average cluster heads within one hop of one sensor node. When a sensor node wants to select a cluster head, the fewer broadcast packets it receives from the neighboring cluster head, the less energy it takes to receive packets. As shown in Figure 11, the average number of cluster heads in one hop using the CNP algorithm is significantly more than that using the PCEB-MS protocol when the number of sinks changes. The number of cluster heads obtained using the CNP algorithm is approximately four when the number of sinks is 12, but this number becomes two when the PCEB-MS protocol is used. Therefore, the probability of cluster interference is reduced by 50%, and, by using the PCEB-MS protocol, fewer sensor nodes are required to decide which cluster they should belong to.

### 6.4. Network Lifetime

**Figure 12:**
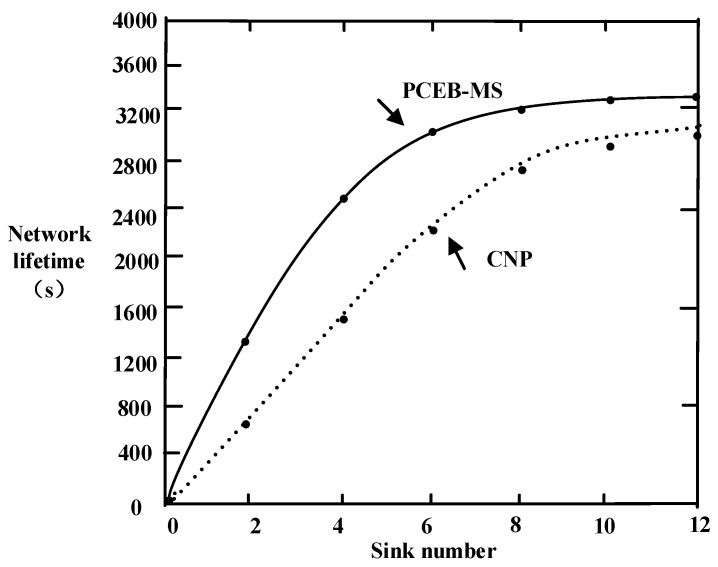
Comparison of network lifetime with different numbers of sinks.

Figure 12 shows a comparison between the PCEB-MS protocol and the CNP algorithm, in terms of network lifetime with an increasing number of sinks, where the average value of the network lifetime is calculated by conducting 50 simulations using both algorithms. The figure shows that the network lifetime of the two algorithms has an increasing trend with an increase in the number of sinks, but there is minimal change observed in the network lifetime when there are more than eight sinks. Using the PCEB-MS protocol, the network lifetime is approximately 3200 s with eight sinks and approximately 3300 s with 12 sinks. However, using the CNP algorithm, the network lifetime is only approximately 2800 s with eight sinks and approximately 3000 s with 12. Therefore, the network lifetime of the CNP algorithm only increases by 14%. It is thus evident that the superior energy balance of the PCEB-MS protocol helps to increase the network lifetime.

### 6.5. Delay

Delay is defined as the end-to-end time. In our study, this refers to the time required for the data packet to be transmitted from the sensor node to the sink. The total delay includes the cluster formation time, the time that the sensor nodes take to send data to the cluster head, and the time that the cluster head takes to transmit data to the sink. An experiment was conducted to analyze the length of the delay that occurs when the number of sinks is increased. The simulation time was set from 0 to 10 min and the data rate created by each source node was 1000 bit/s.

Akkays proposed that packet delay is a very critical factor for the real-time scene [46], and defined delay as the maximum hop (K-hops) count from the network boundary to the sink. With an increase in the hops there will be a greater delay; therefore, an effective method used to shorten delay is to decrease the maximum hop count. In our study, a whole network coverage rate is achieved after the sink adjusts the optimal transmit power; the sink then sends broadcasts to its coverage area, a cluster is formed using the UCEBA algorithm, and all the sensor nodes transmit data to the sink in one hop or two hops. Because the time taken to form the cluster is relatively the same with the number of sinks used, in this experiment we mainly compare the time that the sensor nodes take to send data to the cluster head, and the time that the cluster head takes to transmit data to the sink.

Because the sensor nodes only need to communicate with the cluster head or sink, when more sinks are deployed in the network the data traffic in the network is effectively reduced, and the load of the network greatly decreased. In addition, the average number of hops is reduced, and therefore the length of the delay is eventually improved. 

**Figure 13:**
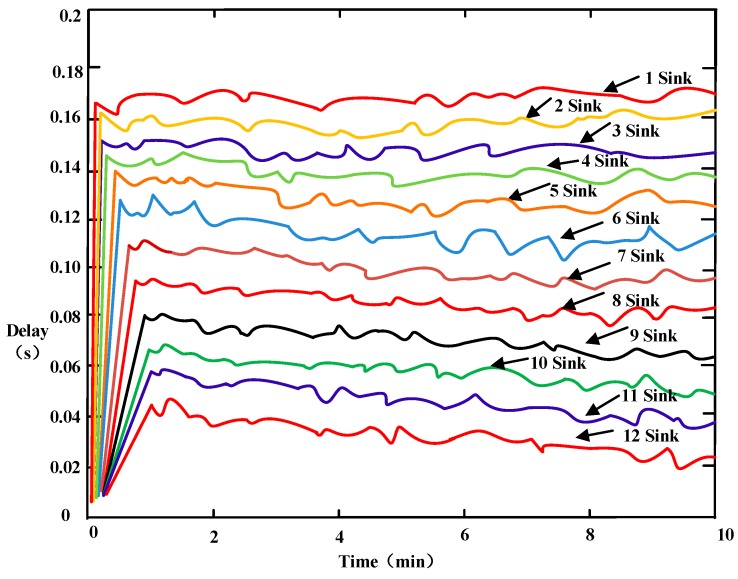
Comparison of data packet delay using varying numbers of sinks.

Figure 13 compares the delay of the data packet with different numbers of sinks used. Delay of the data packet gradually increases over time but is at its lowest when 12 sinks are used. With an increase in the number of sinks, the delay of the data packet increases at a lower level. In addition, with an increase in the number of sinks, the data transmission route becomes shorter and has a lighter load when using the PCEB-MS protocol, thereby significantly reducing network congestion. Therefore, the probability of network congestion and data delay degree is lower when a larger number of sinks are used.

## 7. Conclusions and Future Work

In this paper, the PCEB-MS algorithm is proposed to address problems involved with the use of wireless technology in coalmine tunnels that have a long strip shape. The multisink WSN was used to solve traditional problems existing in underground coalmine tunnels. Network connectivity is improved in relation to power control, and delay is reduced, thereby achieving an energy balance based on network clustering. Results of simulations show that the PCEB-MS algorithm delivers significant improvement to the network performance in terms of power efficiency, connectivity, cluster interference, network life, and delay. We consider that future research should focus on the reasonable deployment of the sink nodes to further improve performance.

## Figures and Tables

**Table 1:** Neighbor nodes of a candidate cluster head.

ID	State	Residual Energy (J)	Distance to Sink (m)
3	Candidate	1.38	10
7	Candidate	0.21	20
8	Candidate	0.15	80
5	Candidate	0.38	60

**Table 2:** Simulation parameters.

Parameters	Values
Network coverage	(0, 0)–(1000, 20) m
Number of sink nodes	1–12
*n*	200
Initial energy of sensor node	0.5 J
*ε*_mp_	0.0013 pJ/bit/m^4^
*E_DF_*	5 nJ/bit/signal
*ε*_fs_	10 pJ/bit/m^2^
*E*_elec_	50 nJ/bit
*t*_0_	4 s
Data grouping	512 bit
*g_t_*	25 dBi
*g_r_*	25 dBi
*λ*	0.1
